# Good and complete responding locally advanced rectal tumors after chemoradiotherapy: where are the residual positive nodes located on restaging MRI?

**DOI:** 10.1007/s00261-016-0640-z

**Published:** 2016-01-27

**Authors:** Luc A. Heijnen, Doenja M. J. Lambregts, Max J. Lahaye, Milou H. Martens, Thiemo J. A. van Nijnatten, Sheng-Xiang Rao, Robert G. Riedl, Jeroen Buijsen, Monique Maas, Geerard L. Beets, Regina G. H. Beets-Tan

**Affiliations:** Department of Radiology, Maastricht University Medical Centre, Maastricht, The Netherlands; Department of Surgery, Maastricht University Medical Centre, Maastricht, The Netherlands; Department of Radiology, The Netherlands Cancer Institute, Amsterdam, The Netherlands; Department of Radiology, Zhongshan Hospital, Fudan University, Shanghai, China; Department of Pathology, Maastricht University Medical Centre, Maastricht, The Netherlands; Department of Radiation Oncology, Maastro Clinic, Maastricht University Medical Center, Maastricht, The Netherlands; Department of Surgery, The Netherlands Cancer Institute, Amsterdam, The Netherlands; GROW School for Oncology and Developmental Biology, Maastricht, The Netherlands

**Keywords:** Rectal cancer, MRI, Lymph node staging, Response assessment

## Abstract

**Purpose:**

Aim of this study was to evaluate the distribution of persistent mesorectal lymph node metastases on restaging MRI in patients with a good or complete response of their primary tumor (ypT0-2) after CRT for locally advanced rectal cancer.

**Methods:**

Two hundred and twenty eight locally advanced rectal cancer patients underwent CRT, which resulted in a good response (downstaging to yT0-2) in 144 patients. Forty-nine patients were excluded (no surgery/insufficient follow-up or lacking lesion-by-lesion histology results). This resulted in a final study group of 95 yT0-2 patients. For the patients with a yN^+^-status, a detailed lesion-by-lesion comparison between restaging MRI and histology was performed to evaluate the characteristics and distribution of the individual N^+^-nodes.

**Results:**

7/95 patients (7%) had a yT0-2N^+^ status (11/880 (1%) N^+^ nodes): no N^+^ were found below the tumor level, 55% of the N^+^ nodes were located at the level of the tumor, and 45% proximal to the tumor (at a median distance of 1.4 cm above the tumor level). In axial plane, 82% of the nodes were located at the ipsilateral circumference of the tumor, at a median distance of 0.9 cm from the tumor/rectal wall.

**Conclusions:**

The incidence of persistent metastatic mesorectal nodes after CRT in patients with a good tumor response after CRT is very low. No N^+^ nodes are found below the tumor level. All N^+^ nodes are located at the level of or proximal to the primary tumor, of which the majority very close to the tumor/lumen.

The staging of lymph nodes remains one of the main diagnostic challenges in the imaging evaluation of rectal cancer. At primary staging, the presence of suspected metastatic lymph nodes can help determine the necessity for neoadjuvant (chemo)radiation treatment (CRT). In patients undergoing neoadjuvant CRT, it has become standard practice in a growing number of centers to perform a restaging MRI 6–8 weeks after CRT to assess the degree of downsizing and downstaging of the primary tumor as well as the nodes. While it is not yet routine practice, organ-preserving treatment (a local excision or ‘wait and see’) is increasingly considered as a potential alternative to standard resection for the very good or complete responding patients after CRT. The first observational trials of ‘wait-and-see’ after CRT have shown promising results regarding long-term survival [1] Habr-Gama et al. recently showed a local recurrence rate of 31% and an overall survival of 91% for patients undergoing a ‘wait-and-see’ policy, with a median follow-up of 5 years [2]. Maas et al. and Smith et al. reported similar results when using strict selection criteria, albeit in smaller patient cohorts [3, 4]. Other groups have performed a local excision (transanal endoscopic microsurgery, TEM) after CRT in patients clinically suspected of having responded well with a small (ycT1-2) residual tumor. For example, Lezoche et al. showed in a randomized trial that a TEM after CRT for patients with a ycT2N0 tumor resulted in a prognostic outcome comparable to TME after CRT [5]. When considering TEM or wait-and-see, an accurate patient selection is an important prerequisite.

Digital rectal examination and endoscopy (with biopsies) are currently the tools most commonly used to assess the local tumor response, because they can evaluate the intraluminal side of the bowel wall. Combined with MRI, these tools have been shown to be highly accurate to identify patients with a complete regression of their primary tumor [6]. DRE and endoscopy are, however, not helpful to assess the mesorectal compartment outside the bowel wall and the nodes within. For this purpose, a restaging MRI could have a particularly beneficial role. Even with a good or complete tumor response the risk for persistent nodal disease is known to be approximately 5%–16% [7–10]. Since the mesorectal compartment—and thus all embedded lymph nodes—remains in situ with both a local excision and ‘wait-and-see’-policy, it is important to identify these nodes. Moreover, knowledge on the distribution of remaining mesorectal nodal metastases could help in understanding which specific areas are at risk to harbor remaining positive nodes and are thus at risk for a nodal tumor regrowth.

The aim of our study is to evaluate the presence and distribution of mesorectal lymph node metastases after neoadjuvant chemoradiotherapy in patients who have responded with a complete or near complete response of their primary tumor, i.e., potential candidates to be considered for organ-preserving treatments.

## Materials and methods

### Patients

All consecutive locally advanced rectal cancer patients treated with neoadjuvant chemoradiotherapy between January 2006 and November 2013 were considered for inclusion in this retrospective study. The study was conducted as part of ongoing clinical studies on nodal imaging, which were approved by the local institutional review board and for which all patients provided written informed consent. Inclusion criteria consisted of (a) biopsy proven rectal cancer with an inferior tumor margin less than 15 cm from the anal verge, (b) age >18 years, (c) long course neoadjuvant treatment, (d) evidence of a good or complete (yT0-2) response of the primary rectal tumor after CRT (either histopathologically proven after TME/TEM or—in case of “wait-and-see” treatment—confirmed by at least 2 years FU and biopsy results without evidence of residual/recurrent tumor or metastatic nodes), and (e) availability of a lesion-by-lesion comparison of lymph nodes between MRI and histology in the patients undergoing resection. Exclusion criteria were (a) pregnancy, (b) non-resectable disease, and (c) contraindications for MRI. In total, 228 consecutive patients underwent long course preoperative chemoradiotherapy. The routine neoadjuvant treatment schedule consisted of 50.4 Gy radiation combined with 2 × 825 mg/m^2^/day capecitabine. Of these patients, 144/228 (63.2%) showed a good or complete response of their primary tumor (yT0-2). Forty-nine were excluded for the following reasons: 42 were treated with organ preservation but did not reach a follow-up period of at least 2 years at the time of writing, 5 were inoperable due to synchronous metastatic disease, and in 2 patients a lesion-by-lesion MR-histological comparison could not be performed. This left a total study population of 95 patients (62 male, 33 female; median age 69 years, range 35–88) with a complete or good tumor response (yT0-2) after CRT.

### MR imaging

All patients underwent a primary staging MRI and a second, restaging MRI routinely performed 6–8 weeks after completion of the preoperative CRT. Patients did not receive bowel preparation. To reduce bowel motility, 20 mg of scopolamine butylbromide (Buscopan, Boehringer Ingelheim, Germany) was administered intravenously just before the MRI, either in case of anticipated bowel movement artifacts on the sagittal planning scan (during the first half of the study period) or routinely (during the second half of the study period). Imaging was performed at a 1.5T MR unit (Intera or Intera Achieva; Philips Medical Systems, Best, The Netherlands) using a phased array body coil. The standard clinical MR protocol consisted of 2D T2-weighted fast spin echo sequences in 3 planes (sagittal, axial, and coronal). Additionally, a 3DT1-weighted gradient echo sequence (TR/TE 9.8/4.6 ms, 15° flip angle, 1 NSA, 1.15 × 1.15 × 1.00 mm voxel size, and 6.30 min acquisition time) was acquired to optimally depict the lymph nodes. The latter sequence was used for nodal evaluation in the current study.

### Image evaluation

An experienced pelvic MR reader (LAH) evaluated each MRI and performed a careful search for all visible lymph nodes within the mesorectal compartment on the 3D-T1weighted GRE sequence. Extramesorectal (lateral) lymph nodes were not taken into account. Multiplanar reformatting of the images was performed to accurately determine the position of each visible lymph node with regard to the primary rectal tumor in three-dimensional plane.

### Lesion-by-lesion MR-histopathological correlation

Similar to the methodology described in previous reports, all nodes visualized on the 3DT1-weighted MRI were drawn on an anatomical map [11, 12]. In the patients undergoing resection, this map was used as a template to ensure accurate node-by-node matching with histopathology. The TME resection specimens were sectioned transversely each 5 mm, perpendicular to the tumor axis. A dedicated pathologist carefully searched for lymph nodes in each section. Each node was placed in a marked individual tray and multiple histological sections were analyzed for each node (for small 1–2 mm nodes only one section was assessed). At microscopic examination, the pathologist reported each node as benign or malignant. For each malignant node, the volume of metastatic disease within the node (%) was estimated. In the patients undergoing a local excision or a ‘wait-and-see’ policy with a recurrence-free follow-up period of 2 years, all nodes visualized on MRI were considered benign lymph nodes.

### Localization of lymph nodes

**Fig. 1:**
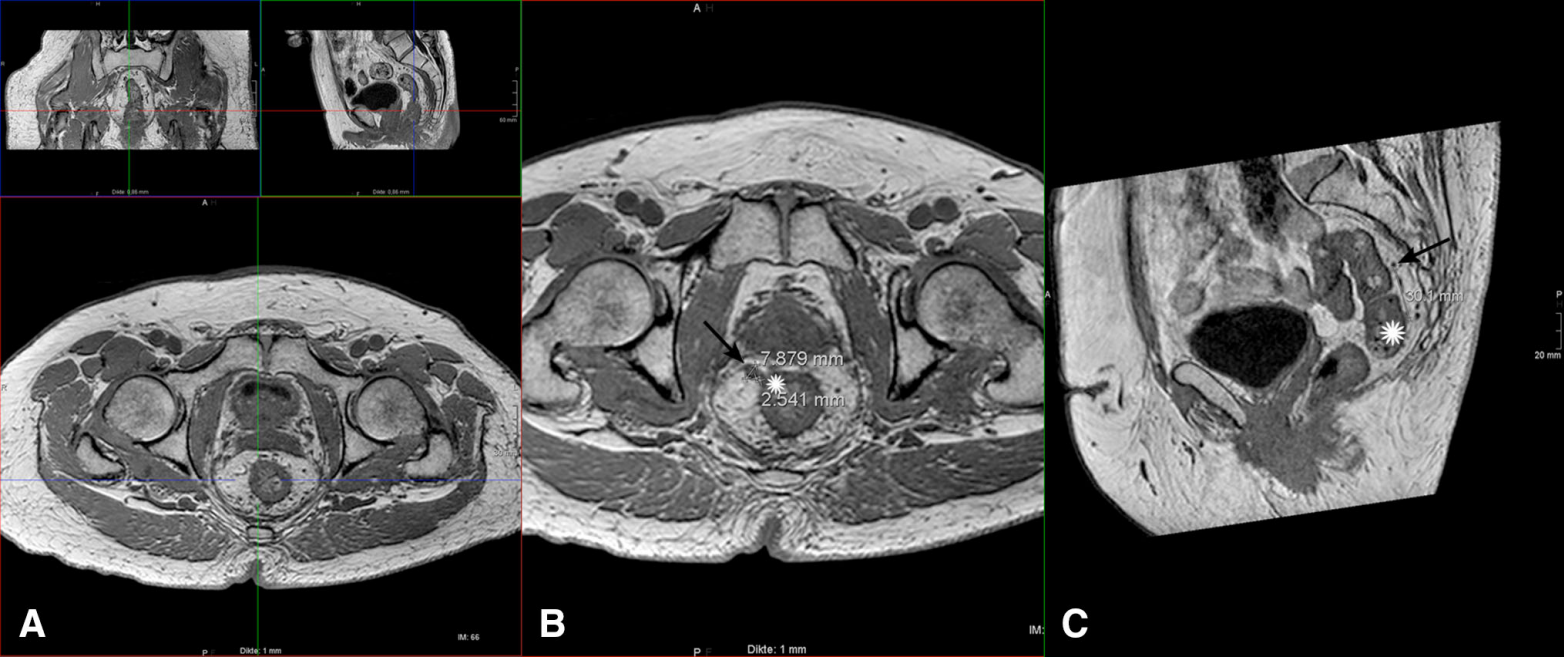
Multiplanar reformatting (MPR) view of the 3D-T1weighted GRE images of a patient with a good tumor response after CRT, with (**A**) an overview of the MPR window, (**B**) the reconstructed axial view, and (**C**) the reconstructed sagittal view. The position of each visible lymph node (*arrow*) with regard to the tumor bed (*asterisk*) was measured in three-dimensional planes

For each individual lymph node depicted on the 3D-T1W GRE sequence (including MPR reformatting), the following parameters were recorded: [1] N^+^ or N^−^ node (based on histopathology and/or long-term FU), [2] size (short axis diameter in mm), [3] position relative to the primary tumor as determined on sagittal or coronal reconstructed plane (i.e., at the level of the tumor, or ×mm distal or proximal to the primary tumor), [4] distance from the primary tumor in axial plane (mm), [5] location (…o’clock) of the lymph node in axial plane/supine position (with 12 o’clock being anterior and 3 o’clock being left lateral location). The nodes localization procedure is illustrated in Fig. 1.

## Results

### Patient characteristics

Patient characteristics are provided in detail in Table 1. Of the 95 study patients, 61 underwent TME and 34 underwent organ-preserving treatment (4 TEM and 30 a wait-and-see policy) with >2 year follow-up. Fifty-four patients had a yT0, 10 a yT1, and 31 a yT2 status. The overall incidence of yT0-2N^+^ disease was 7/95 (7.4%): 3/54 (5.6%) in patients with a complete tumor response (yT0) and 4/41 (9.8%) in patients with a good tumor response (yT1-2). In the 61 patients who underwent a total mesorectal excision, a total number of 671 nodes was harvested from the mesorectal fat at histopathology (median 12 nodes per patient, range 1–25, 660 benign, 11 malignant). In addition, 209 nodes were observed on restaging MRI in the patients undergoing TEM or wait-and see with >2 year FU, and these nodes were all considered benign. Hence, the total number of metastatic nodes after CRT was 11/880 (1.3%).

**Table 1:** Patient characteristics

Features	Number of patients *n* = 95
Gender	
Male	62 (65.3%)
Female	33 (34.7%)
Age (years)	
Median	69
Range	35–88
Tumor height on MRI (measured in cm from anorectal verge)	
Distal (up to 4 cm)	72 (75.8%)
Mid-rectal (from >4 to 8 cm)	19 (20.0%)
Proximal (from >8 to 12 cm)	4 (4.2%)
Length of primary tumor (cm)	
Median	5
Range	1–10
yT stage	
0	54 (56.9%)
1	10 (10.5%)
2	31 (32.6%)
yN stage	
0	88 (92.6%)
1	7 (7.4%)
2	–
Total number of nodes*	
Benign	869
Malignant	11
Number of nodes per patient	
Median	12
Range	1–25
Size non-metastatic nodes (mm)**	
Median	2.9
Range	1.3–18.6
Location non-metastatic nodes	
At the level of the tumor	161 (18.5%)
Distal	47 (5.4%)
Proximal	661 (76.1%)

* 671/880 (76.3%) nodes were confirmed histopathologically, the other 209 nodes (in patients undergoing TEM or wait-and-see) were detected at MRI and confirmed to be negative by >2 year FU without recurrence

** Nodal size was measured on MRI

### Characteristics of the N^+^ nodes

**Fig. 2:**
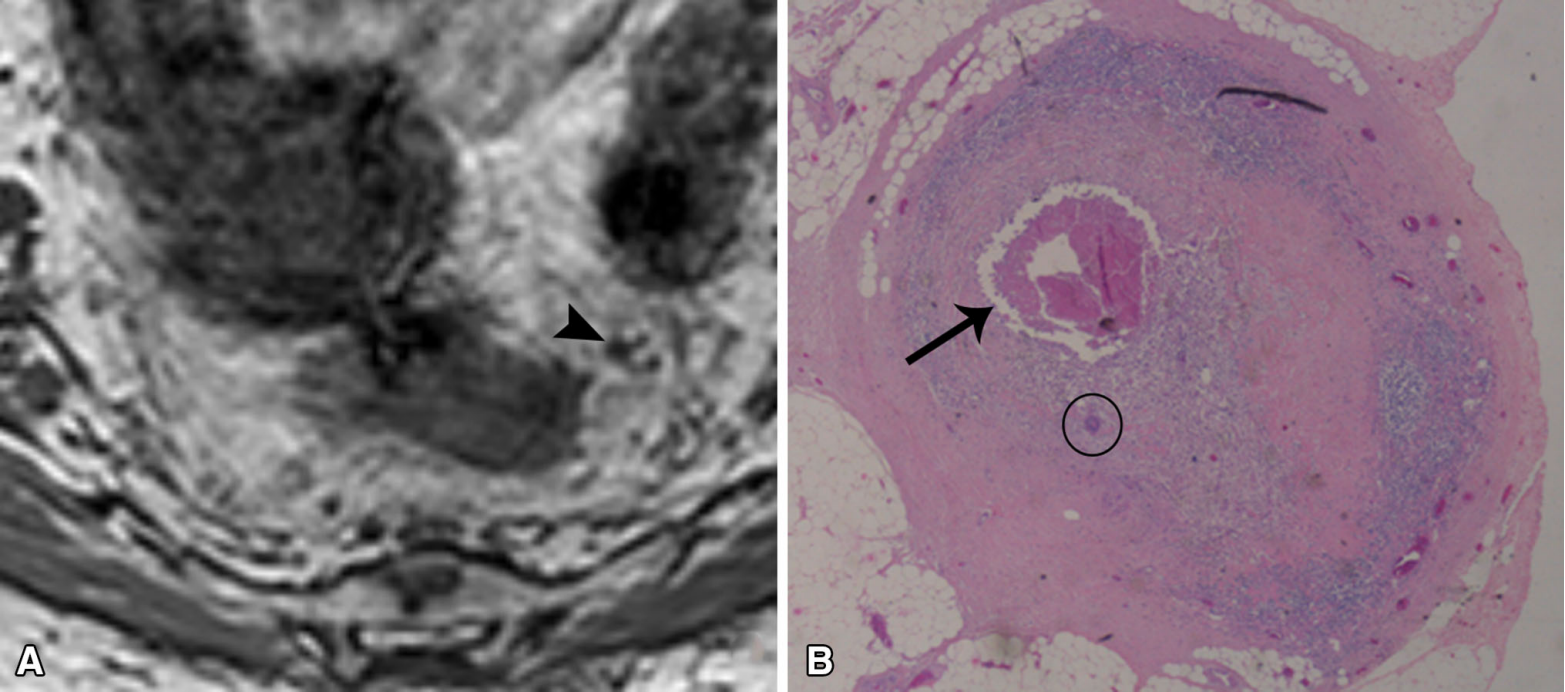
Example of a micrometastasis: **A** 3D-T1W GRE images showed a small (3 mm) node (*arrow*). **B** At histology, a small area of necrosis was present in the center of this node (*arrow*) and only a small cluster of viable metastatic tumor cells (*circle*)

**Fig. 3:**
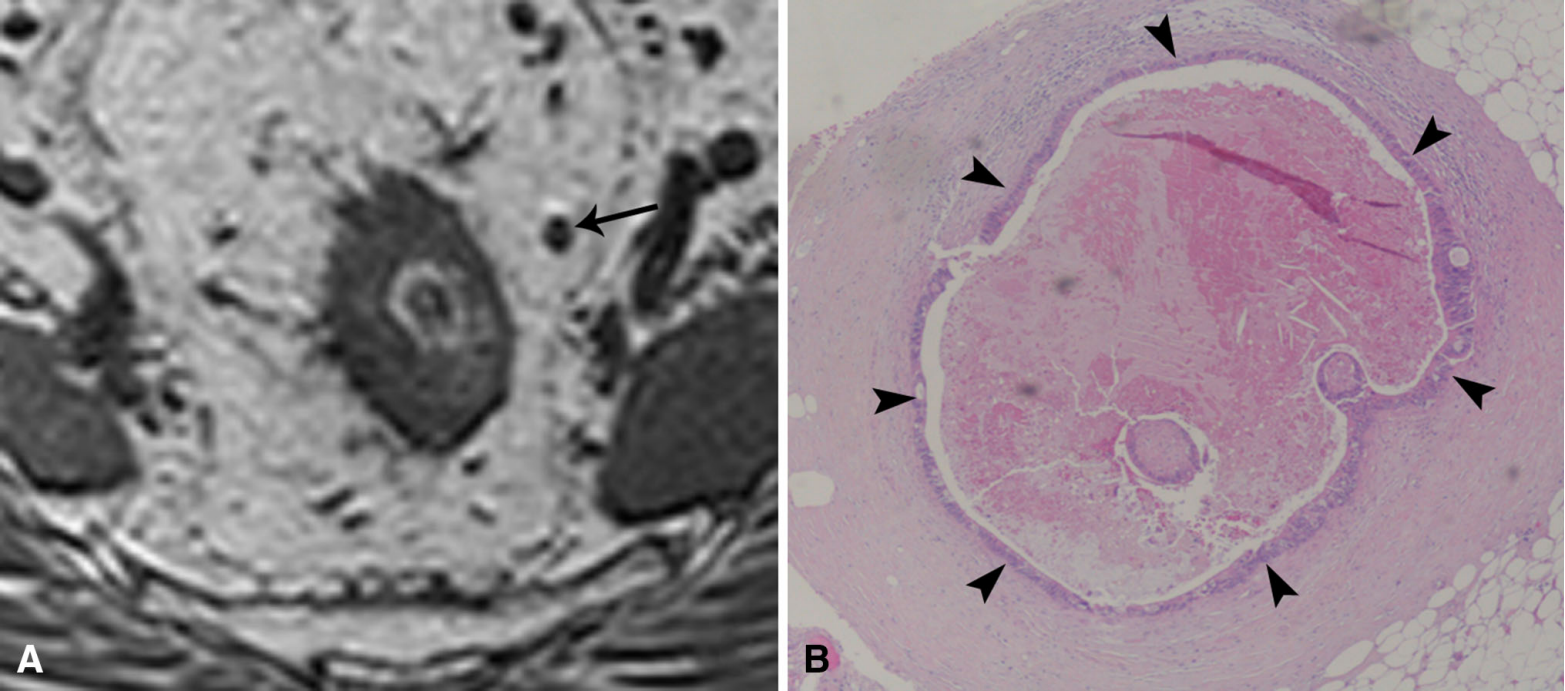
Example of a macro-metastasis: **A** 3D-T1W GRE images showed a 4 mm node (*arrow*). **B** At histology, a large part of the node was necrotic. The necrotic cells were encircled by abundant viable tumor cells (*arrowheads*)

The 11 metastatic nodes were observed in 7 patients. Median size (as measured on MRI) of the non-metastatic nodes was 3 mm (range 1–19 mm), compared to 6 mm (range 3–12 mm) for the metastatic nodes. In the metastatic nodes, the total tumor volume (viable tumor cells ± necrosis) within the node was <25% in 5 nodes (46%), 25%–50% in 1 node (9%), 50%–75% in 3 nodes (27%), and >75% in 2 nodes (18%). Figures 2 and 3 both show an example of a micrometastatic and macrometastatic lymph node, respectively.

### Distribution of lymph nodes

**Fig. 4:**
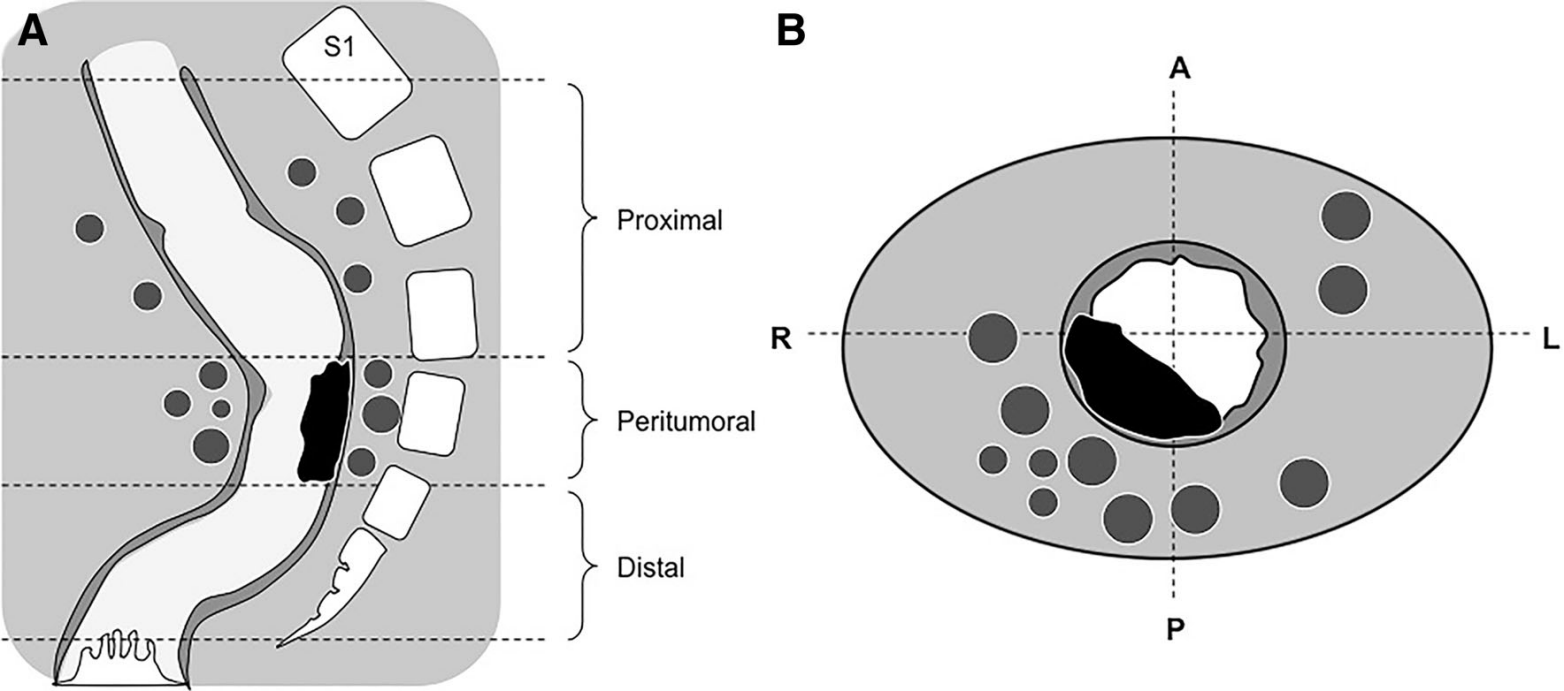
Schematic overview of the location of persistent metastatic lymph nodes after CRT. **A** Distribution of the nodes relative to the residual tumor bed in sagittal plane (proximal, peritumoral or distal). **B** Distribution of the nodes relative to the residual tumor bed in axial plane (ipsilateral or contralateral circumference)

The location of the metastatic lymph nodes in relation to the primary rectal tumor is schematically illustrated in Fig. 4. Further details on the characteristics and location of the individual metastatic lymph nodes are provided in Table 2. No metastatic nodes were found distal to the tumor. Fifty-five per cent of the metastatic nodes were located at the level of the (former) rectal tumor; the other 45% were located proximal to the tumor site at a median distance of 14 mm (10–29 mm) from the most proximal tumor margin. In axial plane, 82% of the metastatic nodes were located at the ipsilateral circumference of the tumor bed and 18% contralateral. The median distance of the metastatic nodes to the tumor bed in axial plane was 9 mm (0–22 mm). Of the non-metastatic nodes 5.4% was located distal to the tumor site, 18.5% was located at the level of the tumor, and 76.1% was located proximal to the tumor.

**Table 2:** Overview and characteristics of metastatic lymph nodes after CRT

Patient	Tumor		Node	Nodal size on MRI (mm)	Volume of metastasis at PA (%)	Axial plane		Sagittal plane
						Distance from tumor (mm)	Position	Distance from tumor (mm)
1	ypT0N1	Mid	1	6	25–50	1	Ipsilateral	0
2	ypT0N1	Distal	2	8	>75	2	Ipsilateral	0
3	ypT0N1	Mid	3	7	<25	9	Ipsilateral	0
4	ypT2N1	Distal	4	8	50–75	0	Ipsilateral	29
5	ypT2N1	Distal	5	12	<25	2	Ipsilateral	18
6	ypT2N1	Distal	6	6	<25	22	Ipsilateral	0
			7	4	<25	9	Contralateral	14
			8	3	<25	13	Contralateral	14
7	ypT2N1	Distal	9	5	50–75	6	Ipsilateral	0
			10	6	50–75	10	Ipsilateral	0
			11	3	>75	10	Ipsilateral	10
			Mean	6		8		8
			SD	3		6		10

## Discussion

The results of our study show that the overall incidence of persistent positive lymph nodes in patients with locally advanced rectal tumors that have responded well and downstaged to yT0-2 after preoperative CRT is 7%; 6% for the complete responders (T0) and 10% for the patients with a small T1-2 residual tumor. No metastatic nodes were found distal to the tumor level; all persistent metastatic nodes were located at the level or proximal to the (former) tumor bed. Furthermore, the metastatic nodes were mainly located in the proximity of the tumor at the ipsilateral circumference.

Ex-vivo cadaver studies have shown that the overall anatomical presence of lymph nodes (in non-oncological patients) is low in the distal mesorectum compared with their presence in the proximal two-thirds of the mesorectum [13]. Furthermore, in patients with rectal cancer, Engelen et al. showed that on primary staging MRI only 10% of the non-metastatic nodes and only 2% of the metastatic nodes were located below the tumor bed, while all other metastatic nodes were located at the same height (66%) or proximal to the tumor (32%) [14]. Koh and colleagues reported similar numbers. They found in a small group of 16 patients that almost all nodes (98%, both benign and malignant) on primary staging MRI were located at the level of or within 5 cm proximal to the tumor [15]. Similarly, in a subsequent small study by the same group on restaging MRI after CRT, it was found that no nodes remained after CRT distal to the tumor [16]. Sprenger et al. confirmed this observation by showing that—after CRT—only 9% of the nodes, regardless of their histopathological features, was found distal to the tumor [17]. Our current study in a significantly larger patient cohort confirmed previous literature: all persistent metastatic nodes were located at the tumor level or above and no metastatic nodes occurred distal to the tumor level. Similarly, of the non-metastatic nodes only 5.4% was located below the tumor level, while the other non-metastatic nodes were all situated at or above the tumor level.

This study specifically focused on the subgroup of patients who showed a complete or near complete response of their primary tumor after CRT. These patients are of particular clinical interest, as they constitute the subgroup that might be considered for organ-preserving treatments. An important topic of ongoing debate is how to select the right patients for organ-preserving treatments without putting the patient at risk for undertreatment and consequently tumor regrowth. A prerequisite for organ preservation is that the primary tumor shows a clinical complete or near complete response and all initially suspicious nodes are sterilized. In a recent report, Perez and co-authors reported on changes in nodal size after CRT in patients with a good primary tumor response [18]. They evaluated a total number of 201 nodes in 31 patients with a ypT0-2 status after CRT and found that metastatic foci were still present in 12/201 (6%) nodes, similar to the observed small number of metastatic nodes (1%; 11/880) in our current report. At the optimal size cut off of ≥4.5 mm, the specificity for assessment of ypN+ disease was high (95%), at the expense of a sensitivity of only 42%. Although it is generally known that size is a poor predictor for rectal cancer lymph nodes, some studies have suggested that size criteria work better in the restaging setting because nodes are smaller in size due to downsizing as a result of the irradiation. Nodes that remain large after CRT are thus more likely to be malignant. Nevertheless, accuracies for nodal restaging with MRI showed a wide range from 67% to 90%, because of the difficulties in detecting and assessing small sized nodes [12, 16, 19–23].

Apart from the identification of positive nodes after CRT, information on the distribution of these nodes could have clinical value. First, it is important to know that no metastatic nodes remain distal to the tumor site to determine the distal resection margin in case of a low anterior resection. When considering a local excision, some authors have suggested that nodes in the proximity of the tumor remnant/rectal wall may be excised with an extended local excision [24]. Furthermore, information on the distribution of remaining positive nodes after CRT could hypothetically be used to plan a targeted boost of radiotherapy specifically focused on sterilizing these nodes. Finally, the nodal distribution after preoperative CRT has been suggested to withhold prognostic information regarding the risk of distant metastatic tumoral spread [25] as well as overall survival [17]. Leibold et al. reported that patients with proximal lymph node metastases (i.e., along major supplying blood vessels) had a significantly higher risk for distant metastases compared with patients without proximal node involvement (46% vs. 25%, *p* < 0.001) [25] Similarly, Sprenger and colleagues found that patients with metastases in proximal lymph nodes after CRT had significantly impaired cancer-specific survival compared to patients with peritumoral nodal metastases only (*p* < 0.05) [17].

Interestingly, about half of the persistent metastatic nodes after CRT in our study contained only small clusters or even single-tumoral cells. One can argue whether these micrometastases impact prognosis and consequently whether it is imperative to recognize these micrometastases since they might progressively response and sterilize at longer follow-up as a late effect of (chemo)radiation [26–31]. This is a topic that will need to be addressed by further studies.

There were some limitations to our study design. First, the total number of individual nodal metastases and N^+^ patients in our study cohort is limited, which is inherent to the specific selection of patients with a good tumor response in whom the risk for nodal metastases is known to be low. Second, 34 patients did not undergo TME surgery, but an organ-preserving treatment, due to which histopathological validation of the nodes was not available. However, these patients remained recurrence free for >2 years, which can serve as a surrogate endpoint for a yN0 status. Finally, this study focused specifically on nodes within the mesorectal compartment. As such, extramesorectal (lateral) pelvic lymph nodes were not taken into account.

In conclusion, our study demonstrates that persistent metastatic lymph nodes in the mesorectum are rare in patients with locally advanced rectal cancer who undergo a complete or near complete response after CRT. The metastatic nodes that persist after CRT are located at the level of or proximal to the initial tumor bed and tend to occur on the ipsilateral circumference of and at close distance to the tumor. No persistent metastatic nodes are found below the tumor level. This information may be valuable for clinical selection and follow-up of patients who are considered for organ-preserving treatment strategies after CRT.
